# Editorial: Mechanisms of Innate Neuroprotection

**DOI:** 10.3389/fneur.2016.00080

**Published:** 2016-05-17

**Authors:** Giuseppe Pignataro

**Affiliations:** ^1^Department of Neuroscience, Reproductive Science and Dentistry, Division of Pharmacology, School of Medicine, “Federico II” University of Naples, Naples, Italy

**Keywords:** preconditioning/postconditioning, epigenetic, ionic homeostasis, microRNA, stroke, seizures, Alzheimer’s disease

**The Editorial on the Research Topic**

**Mechanisms of Innate Neuroprotection**

Two endogenous mechanisms of neuroprotection have been recently described: ischemic preconditioning and ischemic postconditioning. In fact, the concept of tolerance has been introduced long time ago and, remaining in the modern era of medicine, in 1927, the Austrian physician Julius Wagner-Juaregg was awarded the Nobel Prize in medicine for his research on pyrotherapy to treat psychiatric disorders. At that time, patients affected by dementia paralytica were preconditioned by the inoculation of malaria parasites (1).

In the present Research Topic, newly characterized mechanisms involved in preconditioning and postconditioning neuroprotection will be discussed in order to provide tools to plan strategies which induce, mimic, or boost these endogenous protective responses.

In particular, this Research Topic discusses the most important mechanisms involved in preconditioning and postconditioning, presenting a series of papers that provide up-to-date, state-of-the-art information on molecular and cellular mechanisms involved in the neuroprotective process elicited by these endogenous neuroprotectant strategies during brain ischemia and other neurological disorders, such as seizure and Alzheimer. In particular, these aspects are faced by tackling multiple angles of this complex phenomenon. In the first part of the book, three chapters are dedicated to the innate immune system and inflammation, giving special attention to the role of microglia (Fumagalli et al.), macrophages (Amantea et al.), and to some important modulators such as NF-kB (Lanzillotta et al.). The core of the Research Topic is dedicated to posttranscriptional modifications occurring during pre- and postconditioning. These aspects are discussed in seven chapters, including reviews (Saugstad; Jimenez-Mateos; Aune et al.), hypothesis and theory paper (Gidday), and original research articles (Reynolds et al.; Meller et al.; Flores-Rodríguez et al.), that highlight the importance of epigenetic modifications and their roles in mediating pre- and postconditioning neuroprotection. The role of non-coding RNA, with particular regards to microRNA, in innate neuroprotection is summarized in two up-to-date papers (Saugstad; Jimenez-Mateos). Experimental manipulation of miRNAs and/or their targets to induce pre- or post-stroke protection is also presented, as well as discussion on miRNA responses to current post-stroke therapies. Finally, in the last two chapters are described cellular mechanisms for neuroprotection, giving a special attention to those proteins involved in ionic homeostasis maintenance. In fact, although the mechanisms through which these two endogenous protective strategies exert their effects are not yet fully understood, recent evidence suggest that the maintenance of ionic homeostasis plays a key role in propagating these neuroprotective phenomena. In this last part of the book, it will be reviewed the role of plasmamembrane transporters and ionic channels involved in the control of ionic homeostasis and taking part to the neuroprotection induced by ischemic preconditioning and postconditioning, with particular regards to the Na^+^/Ca^2+^ exchangers (NCX), the plasma membrane Ca^2+^-ATPase (PMCA), the Na^+^/H^+^ exchange (NHE), the Na^+^/K^+^/2Cl^−^ cotransport (NKCC), and the acid-sensing cation channels (ASICs) (Cuomo et al.). An interesting view from inside organelles is provided in the review by Sisalli et al.

We hope that this Research Topic will stimulate the continuing efforts to understand the cell and physiological mechanisms underlying the origin of endogenous neuroprotective mechanisms. The understanding of the mechanisms involved in the neuroprotection elicited by preconditioning and postconditioning will be clinically relevant for it will contribute to discover new druggable targets in neurodegenerative disorder intervention. Therefore, the data presented here will highlight the capacity of ischemic preconditioning and postconditioning to be of benefit to patients.

## Author Contributions

The author confirms being the sole contributor of this work and approved it for publication.

## Conflict of Interest Statement

The author declares that the research was conducted in the absence of any commercial or financial relationships that could be construed as a potential conflict of interest.
